# Effect of Previous High Glutamine Infusion on Inflammatory Mediators and Mortality in an Acute Pancreatitis Model

**DOI:** 10.1155/2016/4261419

**Published:** 2016-12-14

**Authors:** Ricardo Garib, Priscila Garla, Raquel S. Torrinhas, Ana I. S. Moretti, Marcel C. C. Machado, Dan L. Waitzberg

**Affiliations:** ^1^Department of Gastroenterology, Digestive Surgery Division, University of São Paulo School of Medicine, LIM 35, São Paulo, SP, Brazil; ^2^Food and Nutrition Research Center (NAPAN), University of São Paulo, São Paulo, SP, Brazil; ^3^Department of Clinical Medicine, Clinical Emergency Laboratory, University of São Paulo School of Medicine, LIM 51, São Paulo, SP, Brazil

## Abstract

Parenteral glutamine supplementation in acute inflammatory conditions is controversial. We evaluated the inflammatory and survival responses after parenteral glutamine infusion in sodium taurocholate-induced acute pancreatitis (AP) model. Lewis rats received 1 g/kg parenteral glutamine (*n* = 42), saline (*n* = 44), or no treatment (*n* = 45) for 48 h before AP induction. Blood, lung, and liver samples were collected 2, 12, and 24 h after AP to measure serum cytokines levels and tissue heat shock protein (HSP) expression. From each group, 20 animals were not sacrificed after AP for a 7-day mortality study. Serum cytokine levels did not differ among groups at any time point, but the intragroup analysis over time showed higher interferon-*γ* only in the nontreatment and saline groups at 2 h (versus 12 and 24 h; both *p* ≤ 0.05). The glutamine group exhibited greater lung and liver HSP90 expression than did the nontreatment group at 2 and 12 h, respectively; greater liver HSP90 and HSP70 expression than did the saline group at 12 h; and smaller lung HSP70 and liver HSP90 expression than did the nontreatment group at 24 h (all *p* ≤ 0.019). The 7-day mortality rate did not differ among groups. In experimental AP, pretreatment with parenteral glutamine was safe and improved early inflammatory mediator profiles without affecting mortality.

## 1. Introduction

Glutamine can become essential during hypercatabolic stress and under critical conditions, such as severe trauma, sepsis, inflammatory diseases, and burns [1]. Glutamine is a fuel source for lymphocytes and enterocytes, a substrate for glutathione and heat shock protein (HSP) synthesis, and a potential inhibitory agent for inflammatory cytokine release [2, 3]. These biological properties could contribute to improving gut barrier and lymphocyte function and to attenuate inflammatory responses [4].

In critically ill patients, glutamine supplementation has been suggested to properly support increased cell proliferation rates, gut barrier protection, and inflammatory dysfunction attenuation [5, 6]. The intravenous administration of glutamine can result in its earlier availability for cell use and could be advantageous for the achievement of rapid inflammatory modulation and protection of cells against damage in clinical critical care conditions. However, unexpected harmful effects of parenteral glutamine supply, mainly in patients with multiple organ failure, have been reported recently [7–9].

These observations have challenged the development of new guidelines for safe glutamine supplementation and have made apparent the need for new experimental studies to better understand this nutrient's mechanisms of action in critical illness. Experimental acute pancreatitis (AP) is an effective model for the study of systemic responses that can be applied to test immunomodulatory therapies [10]. The present study aimed to evaluate the impact of previous parenteral glutamine infusion on inflammatory mediator levels and mortality in acute critically ill conditions, using experimental AP as a systemic inflammation-reproducing model.

## 2. Methods

### 2.1. Animals

Adult male isogenic Lewis rats (*n* = 131, 300–350 g) were purchased from the Animal Laboratory of the Multidisciplinary Center for Research in Biological Science (Campinas, São Paulo, Brazil). Prior to the experiment, the animals were adapted for 5 days in metabolic cages at a controlled room temperature (22 ± 25°C) with a 12 h light/dark cycle and free access to water and standard rodent chow (Quimtia®; Nutrilav, Jundiaí, Brazil). All experimental procedures were approved by the Research Ethics Committee of the School of Medicine, University of São Paulo, São Paulo, Brazil.

### 2.2. Intravenous Access

Animals were anesthetized with an intraperitoneal injection of ketamine (Ketamin-S(+)®, 100 mg/kg body weight; Cristália, Itapira, Brazil) and xylazine (Rompum®, 8 mg/kg body weight; Bayer, São Paulo, Brazil). Intravenous access was achieved by jugular central venous catheterization (CVC), according to a standard technique, followed by connection to a swivel apparatus that allowed the animals to have free mobility [11, 12]. After CVC, all animals received 0.9% saline solution infusion for 24 h. After this period, the animals were randomized to receive 48 h intravenous infusion of 6 mL/day 0.9% saline solution (saline group, *n* = 44) or 1 g/kg body weight dipeptide alanyl-glutamine (Dipeptiven® 20%, Fresenius-Kabi, Bad Homburg, Germany; glutamine group, *n* = 42), or no infusion (nontreatment group, *n* = 45). All animals had access to a standard oral diet (AIN-93M) and water ad libitum during this period.

### 2.3. Experimental Acute Pancreatitis

After 72 h intravenous access, all animals were anesthetized with an intraperitoneal injection of 100 mg/kg body weight ketamine (Ketamin-S(+)®, Cristália) and 8 mg/kg body weight xylazine (Rompum®, Bayer). The pancreas was exteriorized through an abdominal incision and the pancreatic duct was catheterized using a 24-gauge angicatheter. AP was then induced by retrograde injection of 0.5 mL 3% sodium taurocholate solution (Sigma Chemical, St Louis, MO, USA), according to a standard technique [13–15]. Following AP induction, 71 animals were sacrificed after proper anesthetization at 2 h (saline group, *n* = 8; glutamine group, *n* = 9; nontreatment group, *n* = 10), 12 h (saline group, *n* = 9; glutamine group, *n* = 6; nontreatment group, *n* = 9), and 24 h (saline group, *n* = 7; glutamine group, *n* = 7; nontreatment group, *n* = 6) by cardiac puncture for blood and tissue (lung and liver) collection, and 60 animals (*n* = 20/group) were kept alive for mortality analysis.

### 2.4. Serum Cytokine Measurement

Blood samples were centrifuged at 1,000 ×g at 4°C for 10 min to obtain serum. Concentrations of cytokines (interleukin- [IL-] 1, IL-2, IL-4, IL-6, IL-10, interferon- [IFN-] *γ*, and tumor necrosis factor- [TNF-] *α*) were assessed in 500 *μ*L serum by multiplex microsphere immunoassays, using a commercial kit for rats (RECYTMAG® 07-65K; Genesis Ltd., MO, USA). Plates were read in a Luminex analyzer (MiraiBio, Alameda, CA, USA), according to the manufacturer's instructions [16].

### 2.5. Heat Shock Protein Measurement

Approximately 100 mg lung tissue and 50 mg liver tissue were pulverized in liquid nitrogen. The material was homogenized in RIPA lysis buffer (100 mM Tris-HCl [pH 7.5], 1% sodium deoxycholate, 1% NP40, 150 mM NaCl, 0.1% sodium dodecyl sulfate [SDS]) plus protease inhibitors (1 mg/mL pepstatin A, 100 mM phenylmethylsulfonyl fluoride). The samples were then centrifuged at 14,000 ×g for 10 min at 4°C. The supernatants were collected and protein concentrations were quantified using the Bradford method (Bio-Rad Laboratories, Hercules, CA, USA).

Protein samples were added to sample buffer (2% SDS, 60 mM Tris [pH 6.8], 5% mercaptoethanol, 0.01% bromophenol blue) and subjected to electrophoresis in a sodium dodecyl sulfate polyacrylamide gel electrophoresis (SDS-PAGE) system (1.5 M Tris-HCl, 10% SDS, 30% bis-acrylamide, 10% ammonia persulfate, and 3,3,5,5-tetramethylethylenediamine). Proteins were then transferred to nitrocellulose membranes using a semidry transfer apparatus (both from Bio-Rad Laboratories). The membranes were incubated in a blocking solution of 5% skim milk in TBST buffer (50 mM Tris buffer [pH 8.0], 100 mM NaCl, and 1% Tween 20) for 1 h at room temperature. Then, they were washed in TBST and incubated with the primary antibody against the protein of interest (HSP polyclonal goat anti-rat®; Santa Cruz Biotechnology, Santa Cruz, CA, USA) overnight at 4°C. Subsequently, the membranes were incubated in a solution containing the peroxidase-conjugated secondary antibody (1 : 1000; Santa Cruz Biotechnology), and Super Signal detection (Pierce, Rockford, IL, USA) was performed. Protein expression was compared by gel densitometry using the ImageJ public domain software created by Wayne Rasband at the US National Institutes of Mental Health, which has been used previously for the determination of HSP70 and HSP90 [13].

### 2.6. Mortality Observation

After AP induction, 20 rats in each group remained under observation for a maximum of 7 days, with access to standard oral diet (AIN-93M) and water ad libitum. The animals were observed individually every 8 h for death registration. Animals that survived until 7 days after AP were sacrificed with an intraperitoneal injection of 80 mg/kg ketamine hydrochloride (Ketamin-S(+)®; Cristália) and 8.0 mg/kg xylazine hydrochloride (Rompum® 2%; Bayer).

### 2.7. Statistical Analysis

All inflammatory variables were compared using the Kruskal–Wallis and Behrens–Fisher tests, as Kolmogorov–Smirnov tests showed that they were not distributed normally. These comparisons were performed between groups at each time point and within groups over time. Mortality and survival data were evaluated by the Fisher test and Kaplan–Meier analysis, respectively. All analyses were based on a 5% level of significance and were performed using SPSS software (ver. 18.0 for Windows; SPSS, Chicago, IL, USA).

## 3. Results

### 3.1. Serum Cytokine Concentrations

**Figure 1:**
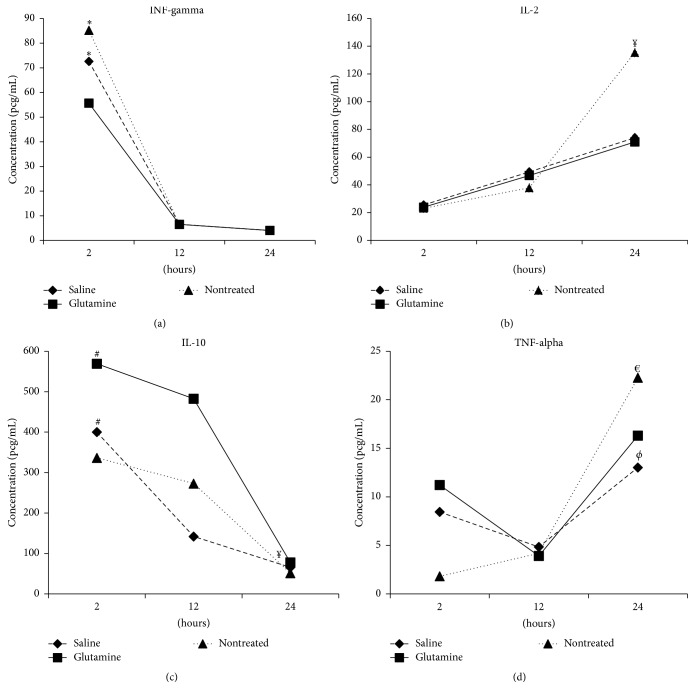
Median serum concentrations of interferon- (IFN-) gamma (a), interleukin- (IL-) 2 (b), IL-10 (c), and tumor necrosis factor- (TNF-) alpha (d) at 2, 12, and 24 h after acute pancreatitis induction with sodium taurocholate in Lewis rats previously treated or not treated with 48 h infusion of parenteral saline or glutamine. ^*∗*^ *p* ≤ 0.050 versus 12 and 24 h; ^*¥*^ *p* ≤ 0.050 versus 2 and 12 h; ^#^ *p* ≤ 0.050 versus 24 h; ^€^ *p* = 0.051 versus 2 and 12 h; ^*ϕ*^ *p* = 0.060 versus 12 h.

Serum cytokine levels did not differ among groups at any time point (Table 1). Serum IFN-*γ* levels in the nontreatment and saline groups were significantly higher at 2 h after AP than at 12 h (*p* = 0.026 and 0.001, resp.) and 24 h (*p* = 0.002 and 0.050, resp.) after AP (Figure 1(a)). In addition, animals in the nontreatment group exhibited higher serum IL-2 levels (Figure 1(b)) and lower serum IL-10 levels (Figure 1(c)) at 24 h after AP than at 2 h (both *p* < 0.001) and 12 h (*p* = 0.005 and *p* < 0.001, resp.) after AP. Animals in the saline and glutamine groups exhibited only lower IL-10 levels at 24 h after AP relative to 2 h after AP (both *p* < 0.001; Figure 1(c)). No significant change in the serum IL-1, IL-4, IL-6, or TNF-*α* level occurred over time, although marginally nonsignificant higher TNF-*α* levels were observed at 24 h after AP in the nontreatment (*p* = 0.051 versus 2 and 12 h after AP) and saline (*p* = 0.060 versus 12 h after AP) groups (Figure 1(d)).

### 3.2. Heat Shock Protein Expression

**Figure 2:**
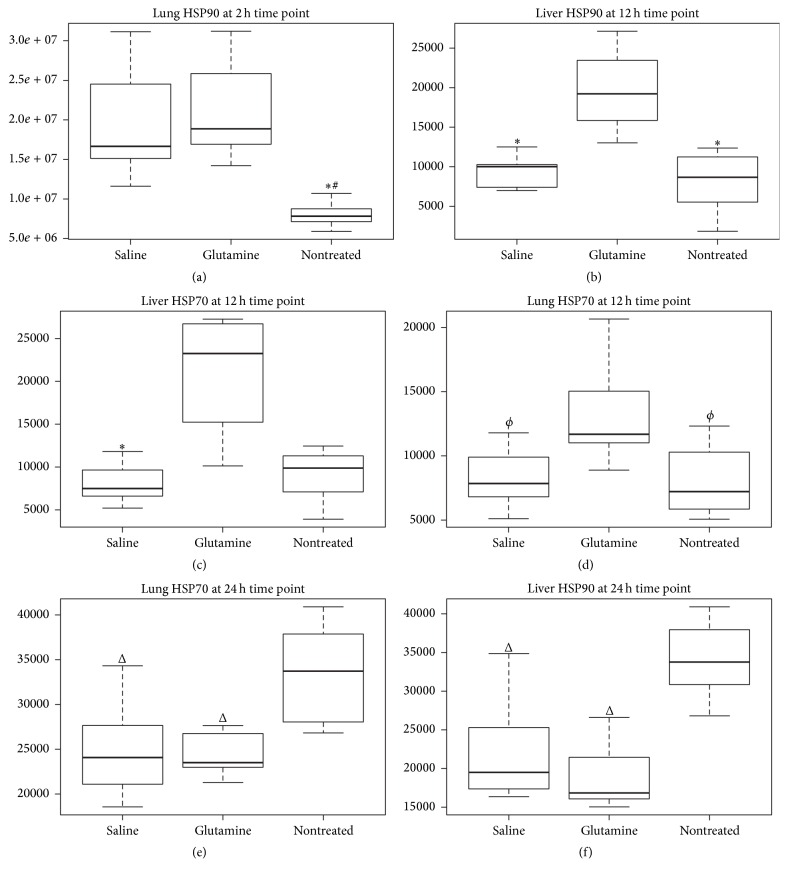
Lung and liver expression of heat shock proteins (HSPs) at different time points after acute pancreatitis induction with sodium taurocholate in Lewis rats previously treated or not treated with 48 h infusion of parenteral saline or glutamine. (a) Expression of HSP90 in lung tissue at 2 h. (b) Expression of HSP90 in liver tissue at 12 h. (c) Expression of HSP70 in liver tissue at 12 h. (d) Expression of HSP70 in lung tissue at 12 h. (e) Expression of HSP70 in lung tissue at 24 h. (f) Expression of HSP90 in liver at 24 h. Data are expressed as medians. ^*∗*^ *p* ≤ 0.050 versus glutamine group; ^#^ *p* ≤ 0.050 versus saline group; ^*ϕ*^ *p* = 0.066 versus glutamine group; ^Δ^ *p* ≤ 0.050 versus nontreatment group.

Data on HSP expression are presented in Table 2. Animals in the glutamine group exhibited greater lung and liver HSP90 expression than did those in the nontreatment group at 2 h (*p* = 0.007; Figure 2(a)) and 12 h (*p* = 0.001; Figure 2(b)) after AP, respectively, and greater liver HSP90 and HSP70 expression than did those in the saline group at 12 h after AP (*p* < 0.001 and *p* = 0.006, respectively; Figures 2(b) and 2(c)). Marginally nonsignificant greater lung HSP70 expression was observed in animals in the glutamine group compared with the other groups at 12 h after AP (*p* = 0.066; Figure 2(d)). Lung HSP90 expression was greater in the saline group than in the nontreatment group at 2 h after AP (*p* = 0.017; Figure 2(a)). The nontreatment group presented increased lung HSP70 and liver HSP90 expression relative to the other groups at 24 h after AP (*p* = 0.019 and 0.004, respectively; Figures 2(e) and 2(f)).

### 3.3. Mortality

**Figure 3:**
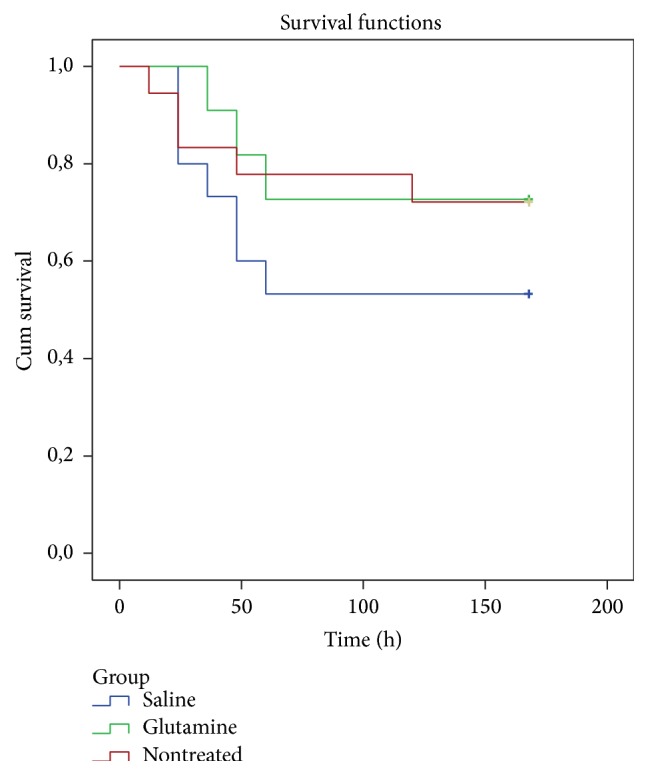
Seven-day mortality rates of Lewis rats treated or not treated with 48 h parenteral infusion of saline or glutamine before acute pancreatitis induction with sodium taurocholate.

No significant difference in 7-day mortality was observed among groups (nontreatment, 28%; saline, 47%; glutamine, 27%) (Figure 3). In addition, the median interval of mortality occurrence did not differ among groups (nontreatment, 24–48 h; saline, 30–36 h; glutamine, 48 h).

## 4. Discussion

Our study aimed to contribute to the understanding of potential inflammatory mechanisms that may impact the risk-benefit balance of parenteral glutamine infusion in critical care. AP was chosen as a critical condition model due to the central roles of inflammatory mediators in its physiopathology and in multiple organ dysfunction syndrome, which is usually its primary cause of death [10]. In addition, the systemic effects of AP are similar to those observed in other critical conditions, such as septicemia, severe burns, and trauma [17]. Specifically, sodium taurocholate-induced AP has been reported to be a representative model of the disease, with severe and measurable systemic inflammatory response and multiple organ failure, as evidenced by lung, liver, and intestinal impairment in rats [18–21].

In human and experimental models, marked release of the proinflammatory mediators IL-1, IL-6, and TNF-*α* is the main detrimental finding associated with AP [15, 22]. This release is usually followed by increased release of anti-inflammatory mediators (e.g., IL-10), which may induce immunosuppression in the late stage of the disease [15]. In our study, high doses of parenteral glutamine infused for 48 h before the induction of experimental AP did not change serum levels of IL-1, IL-6, and TNF-*α* at any post-AP time point. Marginally nonsignificant increases in TNF-*α* levels were observed at 24 h after AP in the nontreatment and saline groups, but not in the glutamine group.

The glutamine group also did not show the significant decreases in IFN-*γ* level observed over time in the nontreatment and saline groups. In addition, the IL-2 levels were maintained overtime in the glutamine and saline groups and the decrease in IL-10 level, observed in all groups at 24 h after AP, occurred more slowly in the glutamine and saline groups than in the nontreatment group. Possible harmful effects and benefits associated with these cytokines must be interpreted in light of the timing of their release over the inflammatory stages of AP progression. For instance, IFN-*γ* and IL-2 can activate inflammation, but these cytokines also have benefits related to pathogen clearance that can be relevant in efforts to avoid infection in the later stages of critical aggression [23, 24].

Accordingly, immunotherapy with IFN-*γ* seems be detrimental in the early stage of AP (when inflammation has harmful effects) and beneficial in the later stage of the disease (when infectious complications and immunoparalysis are dominant causes of mortality) [25–28]. Similarly, decreased IL-2 release and 90% nonspecific mortality were observed after the intraperitoneal administration of lipopolysaccharide in mice with AP, and therapy with recombinant IL-2 reduced lipopolysaccharide-induced mortality in the later stages of the disease [24]. In addition, due to its potent contraregulatory effects, IL-10 has been found to be beneficial in a sodium taurocholate-induced AP model [29]. However, substantial release of this cytokine may hyperintensify its anti-inflammatory effect and favor immunoparalysis [30]. Therefore, the dynamics of IFN-*γ*, IL-2, and IL-10 release over time observed in animals in the glutamine group in this study seem to be protective, enabling the maintenance of immunocompetence for pathogen clearance in the later stages of AP progression.

In our study, marked effects on HSP expression were also observed in the glutamine group in relation to the other groups. This effect included increased liver HSP70 expression and a tendency for increased lung HSP70 expression 12 h after AP, as well as an early significant increase in lung and liver HSP90 expression. Xue et al. [31] reported improvement in the expression of heat shock transcription factor-1 (a master regulator of HSP expression) after parenteral infusion of glutamine in rats. HSP expression may be vital to cellular and tissue protection in the context of stress or injury, as HSPs act as molecular chaperones that stabilize and refold damaged intercellular proteins and prevent intracellular protein aggregation [32]. Indeed, the main metabolic and stress-signaling effects of glutamine in illness and injury seem to occur due its ability to induce HSP expression [33].

Increases in HSP70 expression induced by glutamine are associated with improvements in survival, tissue injury, and inflammatory response [32]. HSP90 also has cytoprotective properties, but most of its target proteins are kinases and transcription factors that can act as cellular regulators of gene expression, including the transcription of proinflammatory molecules via nuclear factor kappa B [34, 35]. However, we found no systemic detrimental increase in proinflammatory cytokines in parallel with increased HSP90 in the glutamine group. Moreover, compared with the nontreatment group, the increased liver HSP90 expression in the glutamine group occurred early after AP induction and was reduced significantly after 24 h. Because HSP expression increases in response to detrimental stimuli, this observation is highly suggestive of early liver homeostasis in response AP injury in the glutamine group [36].

Parenteral glutamine supplementation was recently associated with high mortality rates in critically ill patients with multiple organ failure [7, 8]. With consideration of systemic disturbances that could culminate in multiple organ failure, our AP model did not confirm this harmful effect, despite our parenteral infusion of high glutamine doses. The most recent multicentric trial showed that parenteral glutamine infusion did not change the mortality rate of patients in the surgical intensive care unit but also did not improve clinical outcomes [37]. In our study, the mortality rate was lower and death occurred later in animals in the glutamine group compared with the other groups, although these differences were not significant.

Our study has some limitations in addition to its experimental nature, which may limit the applicability of the findings to humans. First, glutamine was infused alone and before critical stress. As the release of inflammatory mediators is transient, this strategy was adopted to provide glutamine to cells and tissue in time to observe its modulatory effects on these mediators in our model. However, we do not know whether the same effect would be observed if glutamine were infused with other nutrients and in the presence of stress factors. In addition, parenteral supply of glutamine before critical stress cannot be applied fully in clinical practice. Second, saline was used as a parenteral control for glutamine. Saline hydration can attenuate AP by mitigating changes in pancreatic microcirculation and circulatory disorders of the intestinal wall, which facilitate bacterial translocation and perpetuate the inflammation mechanism [38]. These effects may explain the greater benefits of glutamine on systemic proinflammatory mediator profiles and tissue HSP expression in comparison with the nontreatment group than in comparison with the saline group.

Within these limitations, our data suggest that a high dose of parenteral glutamine protects against stress-induced organ damage by improving cytokine profiles and increasing HSP70 and HSP90 expression in our AP model. These protective effects are of particular interest for the treatment of critically ill patients. Further studies must seek to design a protocol for parenteral administration of glutamine that allows us to take clinical advantage of its potential benefits.

## Figures and Tables

**Table 1:** Serum cytokine levels in rats with acute pancreatitis, according to treatment and time point.

Cytokine	Treatment	Time point											
		2 h				12 h				24 h			
		Median	1° quartile	3° quartile	*p* value	Median	1° quartile	3° quartile	*p* value	Median	1° quartile	3° quartile	*p* value
IL-1a	Saline	14.41	4.67	47.89	0.552	15.60	11.40	31.86	0.574	1.84	1.84	46.08	0.583
	Glutamine	4.67	4.67	12.27		13.57	9.48	33.94		12.49	1.84	25.98	
	Nontreatment	8.47	4.67	47.89		8.57	6.13	17.86		72.61	1.84	128.00	

IL-4	Saline	3.98	3.98	8.90	0.970	5.91	4.88	9.23	0.464	1.30	1.30	13.10	0.398
	Glutamine	3.98	3.98	3.98		5.91	3.91	15.38		1.30	1.30	4.88	
	Nontreatment	3.98	3.98	3.98		2.17	1.35	6.98		19.72	1.30	42.82	

IL-2	Saline	25.50	22.32	31.21	0.833	49.40	10.97	72.34	0.814	74.03	27.12	117.21	0.249
	Glutamine	23.89	22.31	30.38		46.75	18.16	90.19		70.94	17.49	167.75	
	Non-treatment	23.10	17.69	33.71		37.89	24.44	48.50		135.50	83.31	166.00	

IL-6	Saline	200.00	76.71	289.50	0.644	220.00	50.13	12704.00	0.971	369.00	184.21	766.50	0.458
	Glutamine	178.00	33.11	452.00		331.25	29.70	1619.00		903.00	486.50	1089.00	
	Nontreatment	336.00	33.11	3135.00		1789.00	113.00	5319.00		464.50	293.00	599.00	

IL-10	Saline	400.00	294.50	560.00	0.288	142.00	99.08	691.00	0.964	65.83	34.62	183.18	0.553
	Glutamine	539.00	298.00	893.00		482.50	65.28	1521.00		77.82	54.16	125.41	
	Nontreatment	336.50	168.00	482.00		273.00	96.09	617.00		51.42	39.50	57.66	

IFN-*γ*	Saline	72.62	30.44	89.41	0.142	6.50	6.50	6.50	0.908	4.01	4.01	4.01	0.776
	Glutamine	55.67	55.67	64.15		6.50	6.50	6.50		4.01	4.01	4.01	
	Nontreatment	85.25	64.15	93.61		6.50	6.50	6.50		4.01	4.01	63.08	

TNF-*α*	Saline	8.44	1.82	15.97	0.390	4.84	2.17	5.86	0.862	13.02	9.19	23.27	0.579
	Glutamine	11.22	1.82	11.22		3.91	2.17	8.05		16.31	3.94	18.50	
	Nontreatment	1.82	1.82	8.53		4.19	2.43	9.21		22.27	14.12	38.39	

IL, interleukin; INF, interferon; TNF, tumor necrosis factor.

**Table 2:** Heat shock protein expression in rats with acute pancreatitis, according to treatment and time point.

Variable	Treatment	Time point											
		2 h				12 h				24 h			
		Median	1° quartile	3° quartile	*p* value	Median	1° quartile	3° quartile	*p* value	Median	1° quartile	3° quartile	*p* value
HSP70 lung	Saline	15615447	14285740	20267619	0.372	7848	6817	9901	0.066	24064	21086	27654	**0.0194**
	Glutamine	13645497	8114841	15673669		11684	11021	15036		23499	22976	26745	
	Nontreatment	12956058	10096912	16820669		7219	5854	10299		33718	28039	37863	

HSP90 lung	Saline	16654811	15125912	24525217	**0.009**	8079	6392	9511	0.065	14031	11409	28005	0.1067
	Glutamine	18875083	16923497	25849518		11742	9796	13052		8108	6184	9750	
	Nontreatment	8037912	7573083	12943462		8745	5670	10700		9094	7174	12214	

HSP70 liver	Saline	27154796	22116953	32188418	0.175	7497	6614	9658	**0.027**	24495	20696	24938	0.1495
	Glutamine	28513246	26185806	35924403		23252	15236	26715		15817	13442	20652	
	Nontreatment	34420660	30578261	41040332		9875	7099	11308		20686	14079	21387	

HSP90 liver	Saline	NS	NS	NS	NS	10014	7396	10263	**0.008**	19507	17368	25291	**0.0045**
	Glutamine	NS	NS	NS		19218	15843	23452		16841	16074	21436	
	Nontreatment	NS	NS	NS		8671	5527	11237		337601	30857	37946	

NS, nonsignificant expression to be detected; HSP, heat shock protein.
